# Effects of an assist-as-needed equipped Tenodesis-Induced-Grip Exoskeleton Robot (TIGER) on upper limb function in patients with chronic stroke

**DOI:** 10.1186/s12984-023-01298-2

**Published:** 2024-01-03

**Authors:** Hsiu-Yun Hsu, Chia-Lin Koh, Kang-Chin Yang, Yu-Ching Lin, Chieh-Hsiang Hsu, Fong-Chin Su, Li-Chieh Kuo

**Affiliations:** 1 Department of Physical Medicine and Rehabilitation, National Cheng Kung University Hospital, College of Medicine, National Cheng Kung University, Tainan, Taiwan; 2 Department of Occupational Therapy, College of Medicine, National Cheng Kung University, No.1, University Road, Tainan, 701 Taiwan; 3 Medical Device Innovation Center, National Cheng Kung University, Tainan, Taiwan; 4 Department of Physical Medicine and Rehabilitation, College of Medicine, National Cheng Kung University, Tainan, Taiwan; 5 Department of Biomedical Engineering, College of Engineering, National Cheng Kung University, Tainan, Taiwan

**Keywords:** Stroke, Rehabilitation robot, Tenodesis grip, Assist-as-needed

## Abstract

**Background:**

The original version of the Tenodesis-Induced-Grip Exoskeleton Robot (TIGER) significantly improved the motor and functional performance of the affected upper extremity of chronic stroke patients. The assist-as-needed (AAN) technique in robot-involved therapy is widely favored for promoting patient active involvement, thereby fostering motor recovery. However, the TIGER lacked an AAN control strategy, which limited its use in different clinical applications. The present study aimed to develop and analyze the training effects of an AAN control mode to be integrated into the TIGER, to analyze the impact of baseline patient characteristics and training paradigms on outcomes for individuals with chronic stroke and to compare training effects on the upper limb function between using the AAN-equipped TIGER and using the original prototype.

**Methods:**

This was a single-arm prospective interventional study which was conducted at a university hospital. In addition to 20 min of regular task-specific motor training, each participant completed a 20-min robotic training program consisting of 10 min in the AAN control mode and 10 min in the functional mode. The training sessions took place twice a week for 9 weeks. The primary outcome was the change score of the Fugl–Meyer Assessment of the Upper Extremity (FMA-UE), and the secondary outcomes were the change score of the Box and Blocks Test (BBT), the amount of use (AOU) and quality of movement (QOM) scales of the Motor Activity Log (MAL), the Semmes–Weinstein Monofilament (SWM) test, and the Modified Ashworth Scale (MAS) for fingers and wrist joints. The Generalized Estimating Equations (GEE) and stepwise regression model were used as the statistical analysis methods.

**Results:**

Sixteen chronic stroke patients completed all steps of the study. The time from stroke onset to entry into the trial was 21.7 ± 18.9 months. After completing the training with the AAN-equipped TIGER, they exhibited significant improvements in movement reflected in their total score (pre/post values were 34.6 ± 11.5/38.5 ± 13.4) and all their sub-scores (pre/post values were 21.5 ± 6.0/23.3 ± 6.5, 9.5 ± 6.2/11.3 ± 7.2, and 3.6 ± 1.0/3.9 ± 1.0 for the shoulder, elbow, and forearm sub-category, the wrist and hand sub-category, and the coordination sub-category, respectively) on the FMA-UE (GEE, *p* < 0.05), as well as their scores on the BBT (pre/post values were 5.9 ± 6.5/9.5 ± 10.1; GEE, *p* = 0.004) and the AOU (pre/post values were 0.35 ± 0.50/0.48 ± 0.65; GEE, *p* = 0.02). However, the original TIGER exhibited greater improvements in their performance on the FMA-UE than the participants training with the AAN-equipped TIGER (GEE, *p* = 0.008). The baseline score for the wrist and hand sub-category of the FMA-UE was clearly the best predictor of TIGER-mediated improvements in hand function during the post-treatment assessment (adjusted *R*^*2*^ = 0.282, *p* = 0.001).

**Conclusions:**

This study developed an AAN-equipped TIGER system and demonstrated its potential effects on improving both the function and activity level of the affected upper extremity of patients with stroke. Nevertheless, its training effects were not found to be advantageous to the original prototype. The baseline score for the FMA-UE sub-category of wrist and hand was the best predictor of improvements in hand function after TIGER rehabilitation.

*Clinical trial registration* ClinicalTrials.gov, identifier NCT03713476; date of registration: October19, 2018. https://clinicaltrials.gov/ct2/show/NCT03713476

## Introduction

Stroke is a leading cause of long-term disability resulting from impairments in body structure and function in adults. This situation has created a growing demand for effective approaches to neuro-rehabilitation throughout the world [1]. According to work done in the fields of rehabilitation practice and of experience-dependent neuroplasticity, motor rehabilitation helps chronic stroke patients to recover their motor skills and motor function [2]. However, it has been suggested that the need for effective and accessible interventions for stroke rehabilitation is largely unmet [3].

The application of high-intensity, task-specific regimens to the rehabilitation of stroke survivors shows promise. The beneficial effects of this approach have been attributed to the synaptic plasticity induced by means of exposure to an enriched environment [4], proprioceptive stimulation [5], and motor learning [6]. This approach aligns with the development and utilization of distal hand robotics, potentially offering a targeted and intensified method for restoring hand function in individuals affected by stroke. The goals of this approach are to increase the intensity of the intervention in a controlled manner, as well as reducing the effort required by the therapists to administer repetitive, task-specific training sessions [7]. In comparative studies evaluating the advantages of employing robot-assisted training for both distal and proximal upper extremity (UE), findings have shown that concentrating on distal UE proves more effective in assisting stroke survivors to recover finger motor function [8], muscle strength and quality of movement [9] while performing functional activities. A recent review on robotics' application in hand rehabilitation highlighted that exoskeleton devices offer the advantage of providing passive support or assistance to the distal joints in the hand and wrist. They also offer haptic feedback for training in object manipulation skills. Therefore, the increasing adoption of exoskeletons in distal hand robotics signifies a growing trend [10]. In spite of such promising findings, it has been found that robot-assisted training devices designed to improve functional grasp face certain difficulties due to the need to simultaneously control multiple joints in the hand and wrist [11].

Currently, a variety of exoskeletal devices utilizing different technologies have been proposed. Significantly, their portability makes them a superior choice for use in the rehabilitation of an affected limb [12]. However, a robotic hand-and-wrist exoskeleton must be designed in such a way that its multiple components are exactly aligned with the joints and segments of the affected limb. The fact that the UEs of different people vary widely in their proportions limits the applicability of any given easy-to-use robotic device [11]. In an attempt to address this issue, the Tenodesis-Induced-Grip Exoskeleton Robot (TIGER) [13] was developed based on the concept of functional degrees of freedom (fDOF), taking into account anatomical constraints and incorporating musculotendon routing [14]. The TIGER was equipped with an adaptive actuation mechanism which simplified the multiple DOF of complex hand movements. With the assistance of built-in actuators, the TIGER was used with training paradigms for movements and activity levels which produced significant improvements in the movements of the whole UE as well as of its distal parts. Moreover, the ease with which the TIGER can be set up facilitated its use in clinical setting [15]. However, a major limitation of the original design of the TIGER was its lack of an assist-as-needed (AAN) control strategy designed to provide only the minimum assistance required to complete a target movement.

A number of factors have been found to increase the likelihood that the robot-assisted training of UE function will yield positive clinical outcomes: the characteristics which are specific to certain subgroups of patients [16, 17], the optimal time window for carrying out the training [18], and the types of robots used [19]. However, a recent systematic review of guidelines aimed to identify recommendations for upper limb robotic rehabilitation indicates that the precise patient characteristics benefiting most from this treatment and the optimal timing for its application remain uncertain [20]. Moreover, most of the robotic hand-and-wrist exoskeletons that have been developed so far have only been tested and used in a laboratory setting [21]. Little research has been done to identify the predictors of the effectiveness of using these robotic devices in a clinical setting, thus depriving healthcare professionals of a useful guide to making better clinical decisions. This research gap highlights the need to determine what factors affect the functional outcomes of applying robotic exoskeletons to neuromotor rehabilitation.

The motivation for the current study was addressing this research gap. To this end, our primary objective was to develop a training mode for the TIGER based on an AAN control strategy and to validate the training outcomes for stroke rehabilitation. The design of the AAN-equipped TIGER aligns with neurorehabilitation principles by emphasizing active user participation and engagement. Additionally, it fosters repetitive training to stimulate neuroplasticity and facilitate functional recovery. The secondary objective was to explore the factors affecting training outcomes of using the TIGER to help chronic stroke patients with hemiplegia recover UE function, and the third one was to compare training effects on the motor and functional performance of the affected UE between using the AAN-equipped TIGER and using the original prototype of the TIGER which was designed to provide constant assistance [13]. Although we expected the AAN-equipped TIGER to reduce motor impairment, we hypothesized that the training outcomes would be impacted by the baseline clinical characteristics of the patients and the training paradigms employed.

## Methods

### Design of the AAN control system of the TIGER

**Fig. 1:**
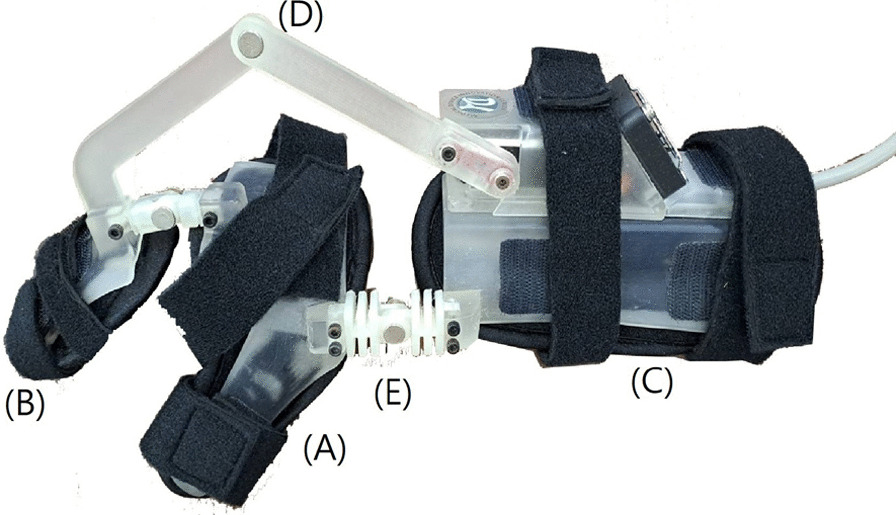
The sideview of the TIGER. The basic parts of the exoskeleton include: a thumb post (**A**), a dorsal shell for the index and long finger (**B**), a forearm trough (**C**), a 4-bar linkage component (**D**), and a resilient lever connected between thumb post and forearm trough (**E**)

As it was originally designed by Hsu et al. in 2021, the TIGER is a robotic exoskeleton linking the hand, wrist, and forearm with one DOF for wrist flexion and extension (Fig. 1). The radial-ulnar deviation along the axis of movement during the flexion–extension of the wrist joint is performed by a resilient lever which connects the thumb post to the forearm post but is not actuated. The wrist motion is driven by a lightweight drone servomotor (SERVOKING DS-685, Taiwan) performing a synergistic grasp-and-release movement with the help of a four-bar linkage mechanism. Utilizing the anatomical arrangement of tendons crossing the wrist joint, when the TIGER induces wrist extension, it tenses the finger flexor tendons, leading to passive finger flexion and facilitating a passive grip function. Conversely, when the TIGER induces wrist flexion, it tenses the finger extensor tendons, aiding in the passive extension of the fingers and assisting in releasing objects held in the hand.

**Fig. 2:**
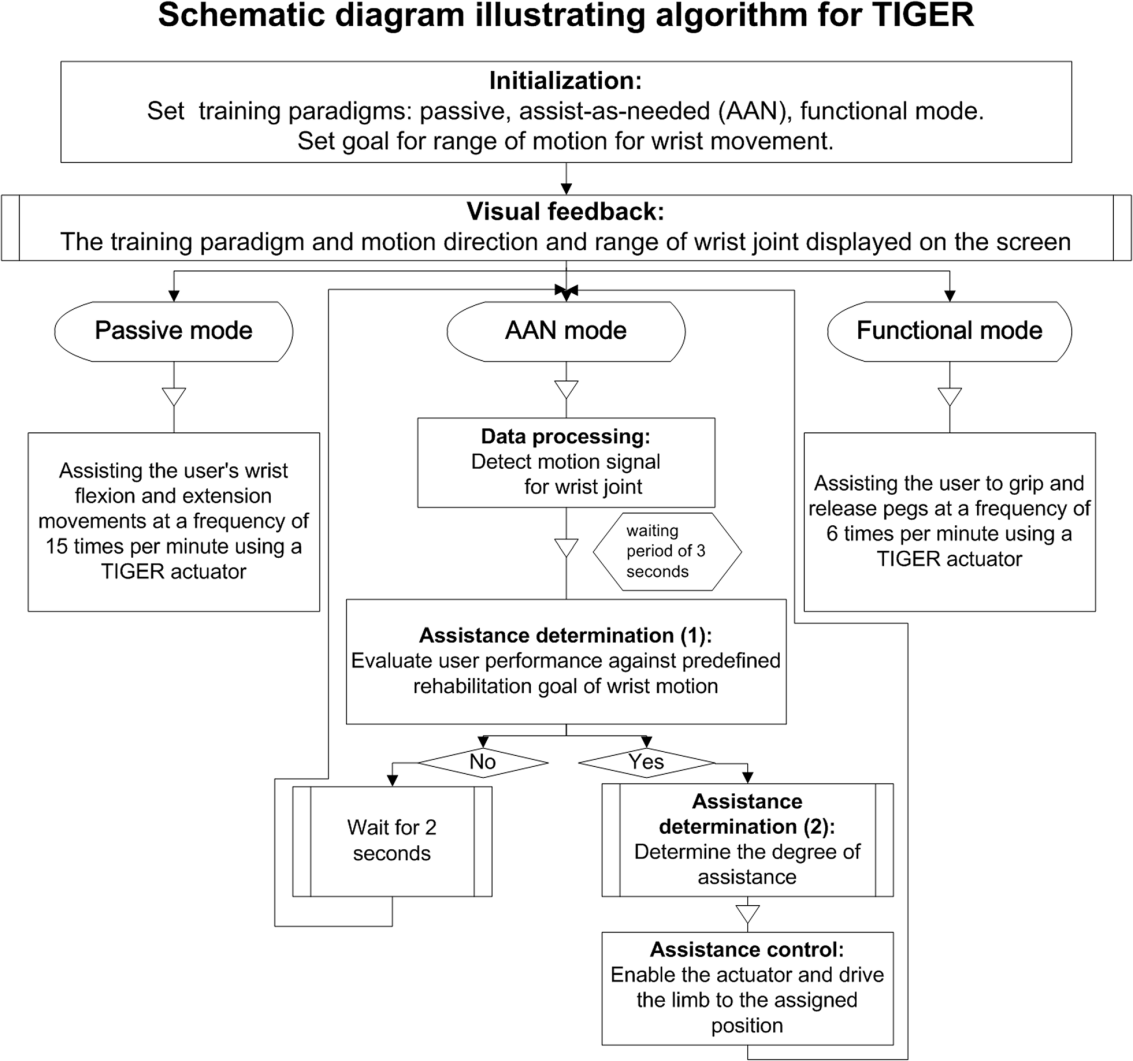
Schematic diagram illustrating algorithm for TIGER

The new version of the TIGER presented in this paper is an attempt to enhance the motor control of the affected wrist and hand by adding a training program based on an AAN control mode. The AAN algorithms consist of three main components: the detection of wrist motion, the modification of the relay feedback tuning method, and the provision of online mechanical assistance for performing wrist movements (Fig. 2). During the training process, a command is displayed on the touch screen prompting the patient to perform a particular motion on their own. The movement trajectories of the affected wrist joint are monitored by means of a current sensor embedded in the control system. If the affected wrist joint fails to reach the specified angle within the allotted time period of 3 s, the AAN-equipped TIGER automatically drives the affected wrist to this angle. The following training paradigms were used to test the new version of the TIGER: a continuous passive motion mode with wrist flexion and extension performed at a frequency of 15 times/min; a functional mode requiring the patient to grip pegs with a Tenodesis grasp and release at a frequency of 6 times/min; and an AAN control mode with movements performed at a frequency of 6 times/min.

### Study design

This is a prospective interventional study with single group design for assessing the feasibility of using the AAN-equipped TIGER to improve the function of a single arm post stroke. Baseline (T1), post-treatment (T2), and follow-up (T3) assessments were carried out by two evaluators, two occupational therapists, and a technician who were blinded to the treatment conditions. The outcomes were compared with the results of using the original TIGER from our previous study [13].

### Participants

The sample size estimation for this exploratory study was derived from our prior TIGER study [13]. Therefore, a minimum of 16 stroke survivors would be recruited to identify the trend or pattern of the treatment effect arising from AAN-equipped TIGER. Suitable participants were identified based on the following inclusion criteria: (1) a disease duration of more than six months post stroke; (2) a score on the FMA-UE of 15–55, which represents an arm-hand motor capacity ranging from none to notable [22]; and (3) adequate cognitive functioning to be able to understand and follow instructions, as represented by a score on the Mini-Mental State Examination (MMSE) of no less than 24. The initial motor scores on the FMA-UE of the participants enrolled in our study on the original version of the TIGER [13] were used as the benchmark for recruiting eligible participants for the current study. The exclusion criteria were: (1) intense wrist pain; (2) noticeable contracture in the wrist and metacarpophalangeal joints; and (3) a score on the Modified Ashworth Scale (MAS) for the wrist and finger flexors greater than 3.

### Intervention

The participants underwent 20-min programs of robotic training followed by 20 min of regular task-specific motor training during each treatment session. More specifically, during each session, participants were trained for 10 min in the AAN mode, which required them to flex and extend their wrist for 60 repetitions, as well as 10 min in the functional mode, when they had to grip pegs for 60 repetitions. In addition to these training programs with the robotic exoskeleton, participants completed three specific tasks designed to help them re-learn specific movements. During this session, they were asked to perform 100–120 repetitions of movements requiring them to reach for, grasp, and release an object. The sessions took place twice a week for 9 weeks.

### Outcome measures

#### 1. Primary outcome measure

Fugl–Meyer Assessment of the Upper Extremity (FMA-UE): The FMA-UE, considered the gold standard in instruments used to evaluate the post-stroke motor recovery of an affected UE [23] with good testing accuracy (the area under curve ranged from 0.61 to 0.70) [24], was used to measure changes in UE motor function following treatment. It consists of 33 items which are rated on the basis of how well participants are able to complete each item. Performance is scored on a three-point ordinal scale ranging from 0 (cannot perform) to 2 (performed completely), for a possible overall score of 66 points. The FMA-UE is divided into 3 sub-categories: (1) the shoulder, elbow and forearm sub-category; (2) the wrist and hand sub-category; and (3) the coordination sub-category. Regarding changes in the performance of chronic stroke patients after undergoing the UE training, the minimal detectable change (MDC) for the FMA-UE as a whole is 5.2 points [25], and it is 1.64 for the sum of the scores on the wrist and hand sub-category [26].

#### 2. Secondary outcome measure

Box and Blocks Test (BBT): The BBT is a clinical tool for measuring unilateral gross manual dexterity in people with motor disabilities. It has been shown to have high test–retest reliability with intraclass correlation coefficient of 0.93 [27]. It consists of a box divided into two halves with a partition in the middle. The goal is to move as many cubes as possible from one side of the partition to the other side. The score on this task is calculated as the total number of blocks moved in 1 min. The value of the minimal detectable change (MDC) for the BBT is 5.5 cubes per minute [28].

Motor Activity Log (MAL): The MAL is a subjective tool for assessing the amount of use (AOU) and the quality of movement (QOM) of an affected UE. It is used in real-life conditions and is based on a semi-structured interview. It has been shown to have construct validity, the Spearman rho value was 0.63 with the Action Research Arm score [29]. The value of the MDC for the MAL is between 0.56 and 1.06 [30].

Modified Ashworth Scale (MAS): The MAS is used to evaluate muscle hypertonia [31] by manually moving a specific joint of the affected limb. It is graded on a six-point scale. It has been shown to have good interrater reliability [32].

Semmes-Weinstein Monofilament (SWM) test: The SWM test provides a quantifiable measure of touch-pressure thresholds. It consists of applying a stimulus to the skin by using precisely calibrated nylon monofilaments. It has been shown that weighted kappa values for inter-rater reliabilities was greater than 0.75 and a strong correlation (r = 0.65) with the stroke impairment assessment set for chronic stroke patients [33]. In the current study, increasing pressure was applied to the volar surface of the affected thumb until the monofilament bent, and it was then maintained for 1.5 seconds. The score was recorded as the thinnest monofilament that the participant was able to detect.

Patient Clinical Global Impressions-Improvements (PCGI-I) scale: PCGI-I was used to gauge subjective feelings regarding the effects of AAN-equipped TIGER on the improvement of the patient’s symptoms. PCGI-I has demonstrated a strong correlation with evaluations provided by clinicians [34]. This scale utilizes a 7-point rating system, ranging from 1 (indicating a significant improvement) to 7 (indicating a considerable worsening of symptoms).

### Protocol for reporting any adverse effects during the study

In this study, an adverse event was defined as any undesirable injury to the skin, ligaments, or muscles of the affected wrist and/or hand joints which was induced while undergoing training with the TIGER and which required a hospital visit.

### Statistical analyses

The SPSS 17.0 software (IBM Corp., Armonk, NY, USA) for Windows was used to perform the statistical analyses. The descriptive statistics were used to describe the raw data, and the results were expressed as the means and standard deviations of the demographic data and of all outcome measures. To address the autocorrelation in repeated measures among participants, we utilized the Generalized Estimating Equations (GEE) model. This model utilized an unstructured variance–covariance matrix and was adjusted by the pre-treatment score to analyze within-group differences for each outcome variable at various time-points during the evaluation.

Forward stepwise regression was performed to identify the predictor variables for improvement in the BBT score during the post-treatment and follow-up assessments, which was defined as the functional outcome of this study. In the regression model, the following variables were defined as potential predictors: the initial motor, sensory and functional status of the affected upper limb, age, gender, and treatment condition. The predictors were added one by one, starting from the most significant variable. The criteria used for selection or removal of variables from the model was based on probability of the F value. The forward–backward selection method was used with the f-to-enter and f-to-remove stopping rule at probability of F at 0.05 and 0.10, respectively. Collinearity of the variables was monitored with variance inflation factor < 10 considered acceptable. Finally, the percentage of variance explained (*R*^*2*^) and the adjusted *R*^*2*^ were calculated as means of quantifying the goodness-of-fit of the predictive model. Statistical significance was set at *p* < 0.05.

The independent-*t* and Chi-square tests were used to compare the data on the disease characteristics and the clinical data of the patients at baseline which were collected during the current study and during our previous study on the original version of the TIGER [13]. Additionally, we employed a GEE model to make comparisons of the effects over time of administering the treatment with the AAN-equipped TIGER and with the original TIGER. Significance was determined at a threshold of *p* < 0.05.

## Results

The current study was undertaken to develop an AAN-equipped TIGER for training hand-and-wrist movement. Its training effect was validated for mild to notable motor impairments exhibited by chronic stroke patients. Seventeen stroke patients participated in the study, one of whom withdrew during the follow-up assessment due to deteriorating health. Therefore, data from 16 patients were included in the comparison of the effects of being trained with the AAN-equipped TIGER with those of being trained with the original TIGER. No significant between-group differences in the demographic and baseline clinical characteristics of the participants in both studies were found (see Table 1). In addition, no harm or adverse events were reported during the interventions.

**Table 1:** Baseline demographic and clinical characteristics of the recruited participants of the current and our previous study [13] of TIGER. (Reprinted by the permission of the Taylor &Francis Ltd)

	Original-TIGER [13] (n = 17)	AAN-TIGER (n = 16)	*P*-value
Demographic data			
Age (yr)	55.5 ± 13.4	56.1 ± 10.2	0.716
Duration following onset (mon)	23.6 ± 15.9	21.7 ± 18.9	0.788
Sex, no. (female: male)	5:12	8:8	0.296
Side of stroke, no. (right: left)	9:8	9:7	1.000
FMA-UE			
Total score	35.1 ± 14.3	34.6 ± 11.5	0.903

Independent-*t* and Chi-square test was used. The level of significance was set at *p* < 0.05

*TIGER* Tenodesis-Induced-Grip Exoskeleton Robot, *AAN* Assist as needed

**Fig. 3:**
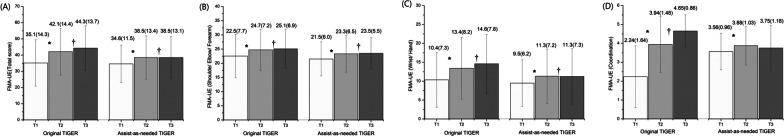
Bar-graph of the sub-score and total score of FMA-UE at baseline (T1), post-treatment (T2) and follow-up (T3) evaluation for the original-TIGER [13] and AAN-TIGER treatment conditions (Reprinted by the permission of the Taylor &Francis Ltd). *Note*: The GEE method was used to detect group by time effects of the two treatment conditions and to detect the within-group difference of variant time-points for each group. The level of significance was set at p < 0.05. *: significant difference within group between the results of baseline (T1) and post-treatment (T2) of treatment. ^†^: significant difference within group between the baseline (T1) and follow-up (T3) results

**Fig. 4:**
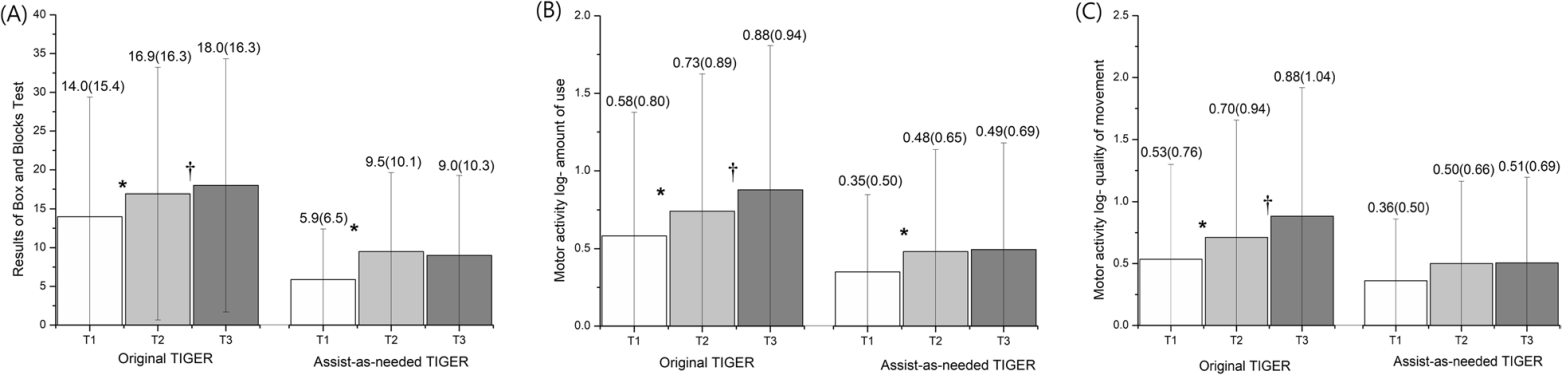
Bar-graph of the functional outcome assessed by Box and Blocks test (**A**) and Motor activity log- amount of use (**B**) and quality of movement (**C**) at T1, T2 and T3 for the original-TIGER and AAN-TIGER treatment conditions (Reprinted by the permission of the Taylor &Francis Ltd)

**Fig. 5:**
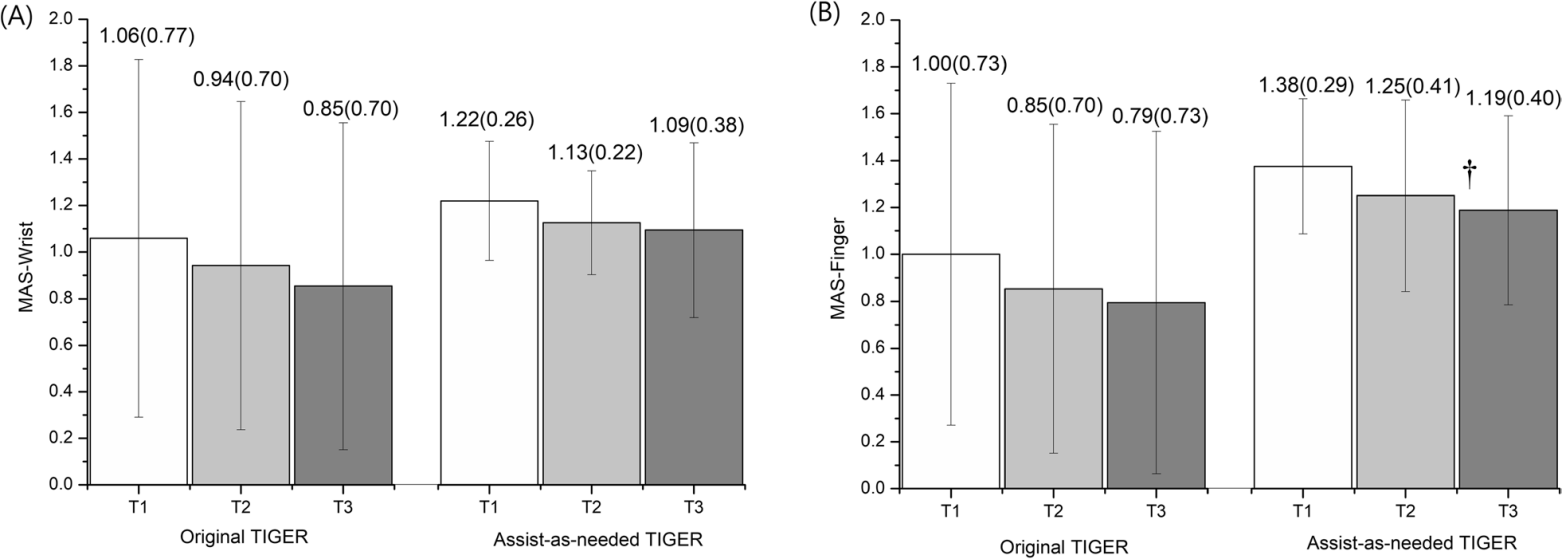
Bar-graph of the assessment of spasticity in wrist (**A**) and finger (**B**) assessed by MAS test at T1, T2 and T3 for the original-TIGER and AAN-TIGER treatment conditions (Reprinted by the permission of the Taylor &Francis Ltd)

Regarding the primary outcome measure, a statistically significant within-group difference (GEE, *p* < 0.05) was found for all scores on the FMA-UE (i.e., the overall score and the scores on all sub-categories) for the participants trained with the AAN-equipped TIGER (see Fig. 3). In addition, the improvement in the score for the sub-category of the wrist and hand reached the CID. Significant improvement was also observed in the functional outcome assessed with the BBT (GEE, p = 0.004) and in the AOU assessed with the MAL (GEE, *p* = 0.02) (Fig. 4). However, the improvements on the BBT did not reach the MDC. Regarding performance on the MAS, finger flexor spasticity showed significant improvement (GEE, *p* = 0.031), whereas this was not the case for the spasticity of the wrist flexor (see Fig. 5). Finally, the SWM score, the measure of sensory function, did not differ significantly among the three time-points after training with the AAN-equipped TIGER (GEE, *p* = 0.443). Alongside the clinical scales, we employed a semi-structured questionnaire, PCGI-I scale, to gauge subjective feelings regarding the effects of TIGER on the improvement of the patient’s symptoms. The mean PCGI-I score was 2.6 ± 1.0 for the AAN-equipped TIGER intervention. Although the PCGI-I scores indicated a positive response from patients towards AAN-TIGER, four patients reported no significant changes in UE movements after the treatment.

The results of undergoing training with the AAN-equipped TIGER were compared with those of training with the original version of the TIGER. A statistically significant group-by-time interaction was found for the total, wrist and hand, and coordination scores on the FMA-UE (see Fig. 3A, C, and D, respectively). The patients using the original TIGER exhibited greater improvements in their performance on the FMA-UE than the participants training with the AAN-equipped TIGER (GEE, *p* = 0.821, *p* = 0.002, *p* < 0.001, and *p* = 0.008 for the scores on the shoulder, elbow, and forearm sub-category, the wrist and hand sub-category, the coordination sub-category, and the FMA-UE as a whole, respectively). Regarding measurements of the functional use of the affected hand, a significant group-by-time effect (*p* = 0.044) was found on the BBT (Fig. 4A). However, the MAL did not reveal any group-by-time effects in terms of the assessment of the AOU and QOM (*p* = 0.314 and *p* = 0.269 for the AOU and QOM, respectively; see Fig. 4B and C). Finally, no significant group-by-time effects were obtained for the score on the SWM test (GEE, *p* = 0.459) or for the MAS scores for fingers (GEE, *p* = 0.984) (see Fig. 5B) and the wrist joint (GEE, *p* = 0.843) (see Fig. 5A).

**Fig. 6:**
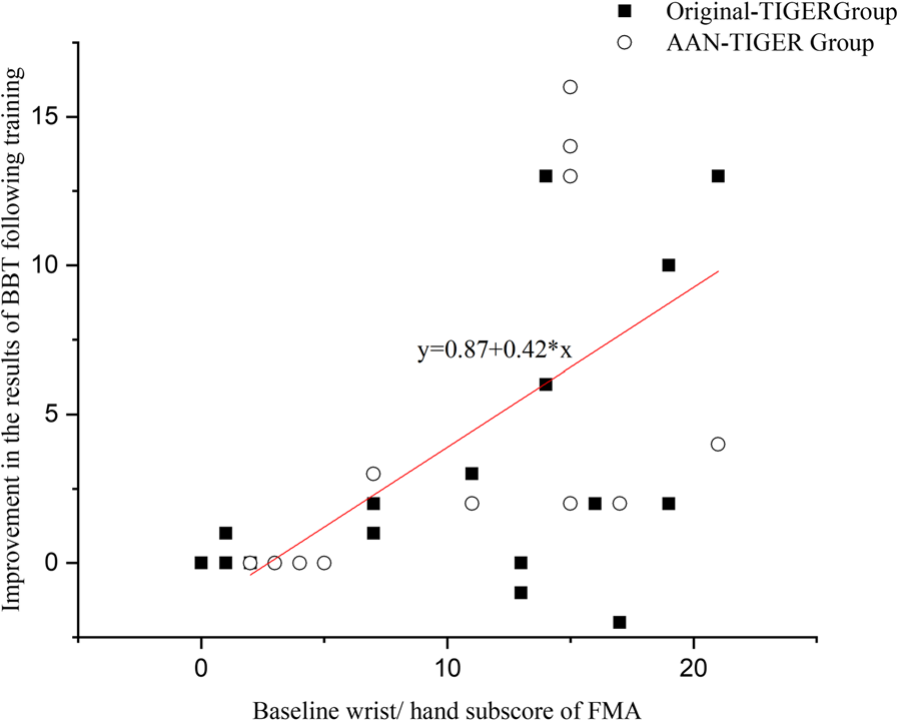
The relationships of baseline wrist/ hand sub-score of Fugl-Meyer Assessment (FMA) and improvement in box and blocks test (BBT) for the TIGER training at post-training evaluation

A multivariate stepwise regression analysis was performed to gain an understanding of the factors that allow predictions of the treatment outcomes for hand function of training with the TIGER. The result indicated that the baseline score for the FMA-UE sub-category of wrist and hand was clearly the best predictor of improvements in hand function during the post-treatment assessment (adjusted *R*^*2*^ = 0.282, *p* = 0.001). The relationship between this baseline wrist and hand sub-score and improvement in the performance on the BBT is shown in Fig. 6. The combination of the baseline characteristics of the wrist and hand sub-score, gender, the BBT score, and sensory status explained 57.2% of the variance of the post-treatment functional outcome for the affected UE (adjusted *R*^*2*^ = 0.572, *p* < 0.001) (Table 2). The training paradigm using the TIGER system did not prove to be a predictor of hand function for the chronic stroke patients in the current study. Finally, the combination of the wrist and hand sub-score and gender accounted for 34.4% of the variance of the follow-up functional outcome for the affected UE (adjusted *R*^*2*^ = 0.344, *p* = 0.001).

**Table 2:** Summarize of forward stepwise regression for gain in BBT at posttreatment and follow-up evaluation

	Model	Predictors	Unstandardized coefficients		Standardized coefficients	*R*^*2*^/adjusted *R*^*2*^	F-value	*P*-value
			Beta	Std. Error	Beta			
Gain in BBT at post-treatment	1	(Constant)	− 0.868	1.348		0.304/0.282	13.544	0.001
		Wrist/hand score of FMA-UE	0.417	0.113	0.551			
	2	(Constant)	4.410	2.365		0.433/0.396	11.476	< 0.001
		Wrist/hand score of FMA-UE	0.507	0.109	0.670			
		Gender	− 3.843	1.468	− 0.379			
	3	(Constant)	3.669	2.176		0.544/0.497	11.542	< 0.001
		Wrist/hand score of FMA-UE	0.735	0.132	0.973			
		Gender	− 3.647	1.341	− 0.360			
		Box and blocks test	− 0.184	0.069	− 0.454			
	4	(Constant)	7.702	2.592		0.625/0.572	11.678	< 0.001
		Wrist/hand score of FMA-UE	0.628	0.129	0.832			
		Gender	− 3.443	1.241	− 0.339			
		Box and blocks test	− 0.207	0.065	− 0.513			
		Sensory status	− 1.261	0.513	− 0.337			
Gain in BBT at follow-up	1	(Constant)	− 0.436	1.430		0.267/0.243	11.300	0.002
		Wrist/hand score of FMA-UE	0.404	0.120	0.517			
	2	(Constant)	4.770	2.548		0.385/0.344	9.389	0.001
		Wrist/hand score of FMA-UE	0.492	0.118	0.630			
		Gender	− 3.791	1.581	− 0.362			

## Discussion

In this study, we developed an AAN-equipped TIGER robotic exoskeleton that offers passive, assist-as-needed, and functional modes for training grasping and releasing movements, and tested its feasibility for clinical applications. The findings indicate that training with the TIGER led to improvements in comparison with the baseline measurements. In particular, motor status improved during the post-treatment and follow-up assessments and functional performance was better during the post-treatment assessment. Regarding the factors affecting the training outcomes, performance on the FMA-UE sub-category of wrist and hand seems to be crucial to determining the effectiveness of the TIGER. Overall, the results supported the two hypotheses of this study.

Our obtained results were in line with the findings of a recent research that an arm robotic exoskeleton system promoted motor recovery of upper extremity in patients with stroke [35]. The finding that performance on the FMA-UE and hand manipulation was significantly better following training with the AAN-equipped TIGER is consistent with those of previous studies applying an AAN therapeutic approach to the robot-assisted rehabilitation [36, 37]. While the FMA-UE gain of only 3.9 points was lower than the previously mentioned minimal detectable change (MDC) of 5.2 points in the Methods section, it surpasses the MDC mentioned in another study, which was 8% of the highest possible score [38] (2.8 points). Furthermore, there was an improvement of 1.8 points in the scores of the wrist and hand sub-category, which exceeded the MDC value. Despite 7 out of 16 participants being characterized as having severe impairments (baseline FMA-UE score 0–30 [39]), one factor contributing to a lesser improvement in training progression [40], these participants also demonstrated meaningful improvements in upper extremity (UE) performance. The AAN-equipped TIGER proved capable of detecting wrist motion while voluntary muscles were contracted, in response to which real-time mechanical assistance was provided to help patients complete the desired wrist motion when the affected wrist joint failed to reach the specified angle. It must be emphasized that, rather than assisting the fingers to move individually, the TIGER was specifically designed to train the affected hand of a stroke patient to perform grasping and releasing movements. To this end, the link between the hand, wrist, and forearm formed by the device exploits the wrist Tenodesis effect in order to control the wrist joints and help the hand open and close the thumb and fingers. By yielding positive results regarding the ability of our participants to use their affected hand to handle objects, this repetitive, active movement-based training technique is in agreement with the previous finding that a device produced the improvement of the upper limb motor function of chronic stroke patients [41].

The performance as assessed by the shoulder, elbow, and forearm sub-category of the FMA-UE after training with the AAN-equipped TIGER was similar to that following training with the original version of the TIGER. However, training with the original TIGER led to better group-by-time effects on the primary outcome of the total FMA-UE score and on the scores for the wrist and hand and coordination sub-categories. This unexpected finding may be attributable to the fact that 5 out of the 16 participants in the current study were incapable of moving their affected wrist at the stage of the baseline assessment. Among the four patients who lacked wrist movement, no significant changes in upper extremity (UE) movements were reported following the treatment, as assessed by the semi-structured PCGI-I scores. This initial condition likely attenuated the expected training effect given that the AAN control mode was designed to provide assistance while the participants moved the robotic exoskeleton on their own using their limited range of motion. In contrast, the participants in the study examining the use of the original version of the TIGER were required to perform 150 repetitions per session of a continuous passive motion of the wrist. This procedure gave them much more practice performing the target movement than was the case for the training conducted with the AAN-equipped TIGER. Therefore, the greater degree of recovery of UE motor function observed with the original TIGER may simply be an instance of the Hebbian theory of plasticity at work [42].

The goal of UE training should be to enable stroke survivors to perform functional movements such as reach-to-grasp [43]. Our research results show that both versions of the TIGER enhance the functional performance of the UE. However, the treatment mode was not a core factor affecting functional outcomes for the affected UE of chronic stroke patients who underwent training wearing a robotic exoskeleton on the hand and wrist. The best regression model in terms of the efficacy of training with the TIGER system incorporated the baseline score for the wrist and hand sub-category of the FMA-UE, the scores on the BBT and the SWM test, and gender, which together explained 57.2% of the improvements in functional performance. In addition, the best single predictor of these improvements was the wrist and hand sub-score, which explained 28.2% of the variance. These results suggest that the baseline assessment of the movement of the wrist and hand is important for predicting the functional recovery of stroke patients with mild to severe initial motor impairment by means of training with a robotic exoskeleton. The obtained finding was supported by a previous study that better hand movement was associated with upper limb motor recovery after robot-assisted upper limb rehabilitation [18]. Moreover, combining the sensory and functional capabilities of the UE assessed at the baseline stage accounted for an even greater proportion of the variance in the improvements in functional performance, which suggests that the sensorimotor function of the UE directly predicts people’s levels of activity post stroke. This conclusion is supported by the recent finding that deficits in body functions and structures impact the improvement of people’s performance [44].

Despite the several contributions of this study, it also had some limitations. First, it did not have a randomized control trial design, which precluded making more specific comparisons of the training effects between the passive mode and AAN control mode of the TIGER. Another limitation is that a 10-min AAN setting might not be adequate to generate significant results. It is essential to perform a thorough assessment to ascertain the optimal therapy regimen, considering diverse frequencies, durations, and potential target populations for our future research. Additionally, the predetermined 3-s waiting time for each movement assistance within the AAN setting might be perceived as lengthy by certain patients. Subsequent versions could explore incorporating a variable or adjustable waiting period to better accommodate individual patient requirements. Furthermore, it would be interesting for researchers to record wrist trajectories in real time as the motion data to design appropriate training paradigms for patients using the TIGER.

## Conclusion

This study presented a new generation of TIGER for post-stroke rehabilitation. The findings of the current study validated the training effects of the AAN-equipped TIGER for use with chronic stroke patients. By integrating the AAN control mode into the original TIGER system, the characteristics of assisting motion as needed, performing continuous passive motion, and carrying out functional training by gripping pegs were embedded in the TIGER. Notably, the initial FMA-UE sub-score for the wrist and hand emerged as the most dependable predictor of improvements in hand function following TIGER rehabilitation. This insight aids in tailoring specific upper extremity (UE) training approaches based on individual motor abilities. However, in terms of the performance and functional use of the affected hand, the AAN-equipped TIGER did not produce superior outcomes compared to original prototype.

## Data Availability

Not applicable.
